# Human Infections with Novel Reassortant Influenza A(H3N2)v Viruses, United States, 2011

**DOI:** 10.3201/eid1805.111922

**Published:** 2012-05

**Authors:** Stephen Lindstrom, Rebecca Garten, Amanda Balish, Bo Shu, Shannon Emery, LaShondra Berman, Nathelia Barnes, Katrina Sleeman, Larisa Gubareva, Julie Villanueva, Alexander Klimov

**Affiliations:** Centers for Disease Control and Prevention, Atlanta, Georgia, USA (S. Lindstrom, R. Garten, A. Balish, B. Shu, S. Emery, L. Berman, K. Sleeman, L. Gubareva, J. Villanueva, A. Klimov);; Battelle, Atlanta (N. Barnes)

**Keywords:** influenza A(H3N2), swine origin, triple reassortants, viruses, influenza, United States

## Abstract

During July–December 2011, a variant virus, influenza A(H3N2)v, caused 12 human cases of influenza. The virus contained genes originating from swine, avian, and human viruses, including the M gene from influenza A(H1N1)pdm09 virus. Influenza A(H3N2)v viruses were antigenically distinct from seasonal influenza viruses and similar to proposed vaccine virus A/Minnesota/11/2010.

Around the world, cases of human infection with swine-origin influenza viruses have been reported sporadically (*1**–**5*). From 1990 through 2010, a total of 27 cases of human infection with these viruses were confirmed by the US Centers for Disease Control and Prevention (CDC) (*4**,**6*). Of these cases, 21 were caused by triple-reassortant influenza A viruses (13 subtype H1N1, 1 subtype H1N2, and 7 subtype H3N2), which have inherited genes from classical swine, avian, and human influenza viruses. The 2009 influenza pandemic, caused by a variant triple reassortant influenza virus, influenza A(H1N1)pdm09 virus (*7**,**8*), proved that swine influenza viruses (SIVs) can cause widespread infection among humans and result in substantial economic costs. In 2010, an increase in the number of human cases of swine-origin influenza (H3N2) virus infection prompted selection of a candidate vaccine virus of swine origin, A/Minnesota/11/2010 (H3N2)v (*9*).

Systematic surveillance and characterization of novel viruses infecting humans and SIVs in swine are critical for early detection of viruses with pandemic potential. Since 2009, CDC has provided public health laboratories with a real-time reverse transcription PCR (rRT-PCR)–based assay for diagnostic testing for influenza (*10*). This assay enables detection and discrimination of influenza A virus subtypes H1N1, H3N2, and H1N1pdm09 and preliminary identification of triple-reassortant viruses possessing the nucleoprotein gene originating from SIVs.

## The Study

In 2011, public health laboratories in 5 states detected 12 cases of human infection with a novel variant of influenza virus, influenza A(H3N2)v virus, by using the CDC rRT-PCR protocol. Respiratory specimens from these patients were sent to CDC for virus confirmation. History of direct or indirect contact with swine was confirmed for 6 patients. However, swine contact could not be verified for the other 6, suggesting that these infections might have been contracted through limited person-to-person transmission (*11**–**13*) (Table 1). All 12 patients recovered fully from their illness (*10**–**12*).

**Table 1:** Results of analysis of viral RNA isolated from original clinical samples from persons with influenza A(H3N2)v virus infection, United States, 2011*

Influenza virus strain	Contact with swine (*11**–**13*)	Specimen collection date	Specimen type	rRT-PCR–positive results†	Genes sequenced‡
A/Indiana/08/2011	No	Jul 24	NPS	InfA, H3, pdmInfA	Full genome
A/Pennsylvania/09/2011	Indirect	Aug 20	NPS	InfA, H3, pdmInfA	Full PB2, PB1, HA, NP, NA, M, NS, partial PA
A/Pennsylvania/10/2011	Direct	Aug 26	NPS	InfA	Full NS, partial HA, M, NA
A/Pennsylvania/11/2011	Indirect	Aug 25	NPS	InfA, H3, pdmInfA	Full PA, NP, NA, M NS, partial PB2, PB1, HA
A/Maine/06/2011	Direct	Oct 10	NPS	InfA, H3, pdmInfA	Full genome
A/Indiana/10/2011§	Direct	Oct 22	Cell culture	InfA, H3, pdmInfA	Full genome
A/Maine/07/2011	Direct	Oct 24	NPS	InfA	Partial HA, M, NS
A/Iowa/07/2011	No	Nov 14	NPW	InfA, H3, pdmInfA	Full PB2, PB1, PA, NP, NA, M, NS, partial HA
A/Iowa/08/2011	No	Nov 14	NS	InfA, H3, pdmInfA	Full genome
A/Iowa/09/2011	No	Nov 14	NS	InfA, H3, pdmInfA	Full genome
A/West Virginia/06/2011	No	Nov 21	NW	InfA, H3, pdmInfA	Full genome
A/West Virginia/07/2011	No	Dec 07	NPS	InfA	Partial HA, NA, M

*Influenza A(H3N2)v, influenza virus variant identified in humans; rRT-PCR, real-time reverse transcription PCR; NPS, nasopharyngeal swab; PB, polymerase basic protein; HA, hemagglutinin; NP nucleoprotein; NA, neuraminidase; M, matrix protein; NS, nonstructural protein; PA, polymerase acidic protein; NPW, nasopharyngeal wash; NS, nasal swab. †Results obtained by using the Centers for Disease Control and Prevention Human Influenza Virus Real-Time RT-PCR Diagnostic Panel. ‡Sequences available from GenBank and the Technical Appendix Table. §This patient was >18 years of age; all others were <18.

**Figure 1:**
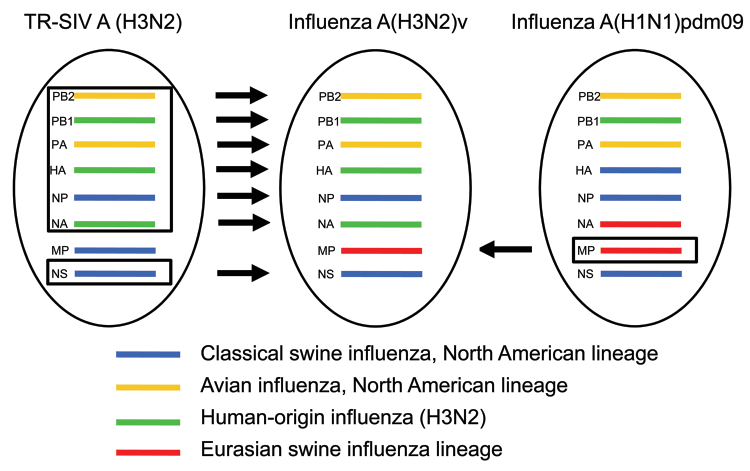
Derivation of genes segments of novel influenza A(H3N2) viruses isolated from humans, United States, 1990–2011. TR-SIV, triple reassortant swine influenza virus.

**Figure 2:**
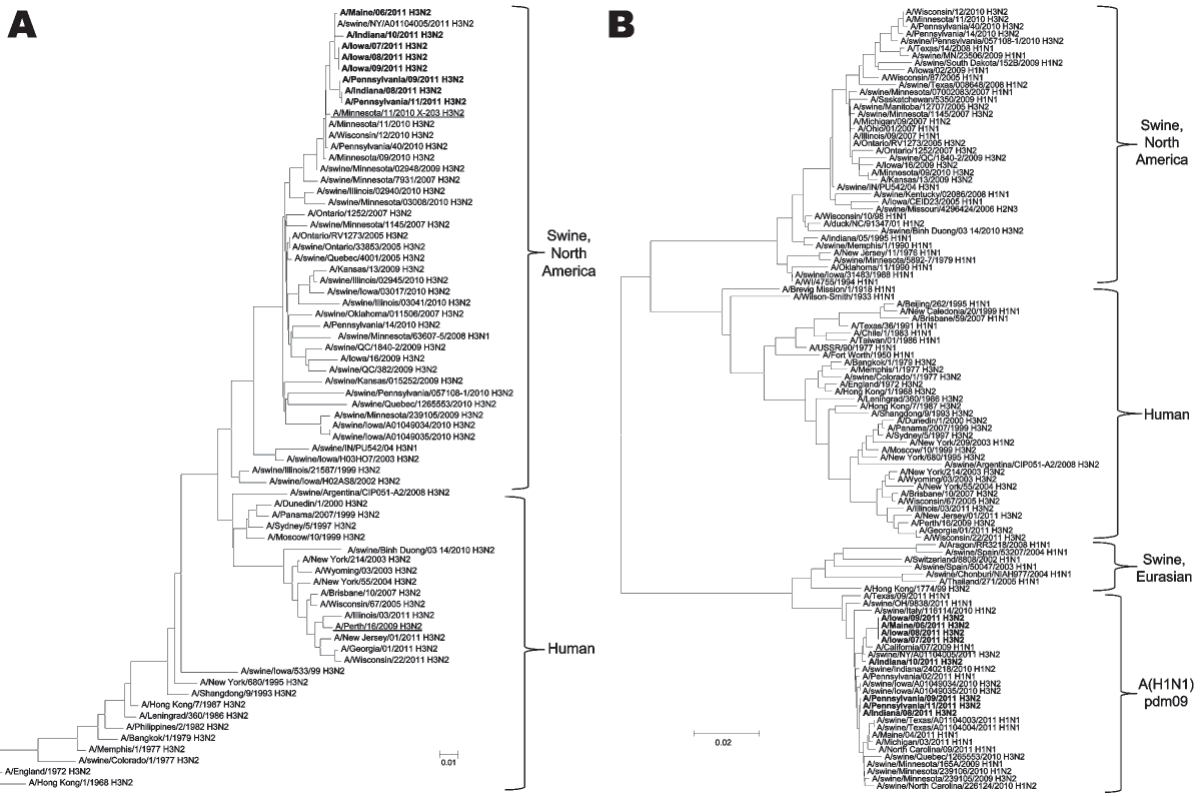
Phylogenetic analysis of the A) hemagglutinin and B) matrix genes of influenza A(H3N2)v viruses. Sequences obtained from human A(H3N2)v isolates in the United States during 2011 are shown in **boldface**; sequences of proposed vaccine virus are underlined. Scale bars indicate number of base substitutions per site.

Genetic sequence analysis of RNA isolated from clinical respiratory specimens (Table 1) revealed that these influenza A(H3N2)v viruses possessed a combination of gene segments not previously found in humans (Figure 1). Of the 8 gene segments, 7 (hemagglutinin, neuraminidase, polymerase basic proteins 1 and 2, polymerase acidic protein, nucleoprotein, and nonstructural protein) were similar to those of triple-reassortant SIV A(H3N2) currently circulating in North America and to those from human triple-reassortant influenza A(H3N2) viruses isolated in 2010 from Pennsylvania, Minnesota, and Wisconsin (*4*), including the proposed vaccine virus of swine origin, A/Minnesota/11/2010 (*14*) (Figure 2, panel A; Technical Appendix Figure). However, the M genes of all 2011 influenza A(H3N2)v viruses were inherited from a pandemic (H1N1) 2009 virus (Figure 2, panel B). Although SIVs of subtypes A(H3N2) and A(H1N2) with the M gene of influenza A(H1N1)pdm09 virus have been detected in swine since 2009 (*15*), influenza A(H3N2)v virus possessing the M gene of influenza A(H1N1)pdm09 virus had not been detected in humans.

According to genetic analysis results, amino acid diversity among influenza A(H3N2)v hemagglutinins was low (0–3 aa) compared with that of influenza A/Minnesota/11/2010. In addition, there have been no conserved amino acid changes in the hemagglutinin when comparing 2011 influenza A(H3N2)v from humans with 2011 influenza A(H3N2) SIVs. In particular, the known receptor binding site of the hemagglutinin protein of influenza A(H3N2)v virus was typical of SIV A(H3N2) viruses recently isolated in North America.

Hemagglutinins of the influenza A(H3N2)v viruses differed substantially from the hemagglutinin of the 2011–12 human seasonal vaccine virus, A/Perth/16/2011 (58–60 aa), which resulted from divergent evolutionary paths for the H3 hemagglutinin in swine and human viruses. The effect of these substitutions on virus antigenicity was examined in the hemagglutination-inhibition assay by using a panel of reference ferret antiserum. Hemagglutination-inhibition analysis of 6 available influenza A(H3N2)v virus isolates revealed no measureable inhibition by antiserum against the current human seasonal influenza A(H3N2) vaccine virus, A/Perth/16/2009 (Table 2), indicating that influenza A(H3N2)v virus is antigenically distinct from influenza A(H3N2) viruses currently circulating among humans.

**Table 2:** Hemagglutinin-inhibition assay results, including the 6 available influenza A(H3N2)v viruses isolated in 2011, United States*

Influenza virus strain (culture method)	Titers to reference ferret antiserum								Specimen collection date
	PER/16	KS/13	WI/12	PA/14	MN/11	X203	IN/08	IN/10	
A/Perth/16/2009 (egg)†	1,280	<10	<10	<10	20	20	<10	<10	2009 Apr 7
A/Kansas/13/2009 (MDCK cells)	<10	640	80	160	40	40	40	80	2009 Jul 29
A/Wisconsin/12/2010 (MDCK cells)	<10	40	1,280	320	640	320	640	1,280	2010 Sep 10
A/Pennsylvania/14/2010 (egg)	<10	160	320	640	320	320	640	640	2010 Oct 26
A/Minnesota/11/2010 (egg)	<10	<10	320	160	640	1,280	320	160	2010 Nov 26
A/Minnesota/11/2010 X-203 (egg)‡	10	<10	80	40	320	640	160	80	Not applicable
A/Indiana/08/2011 (MDCK cells)*	<10	10	1,280	640	640	320	1,280	1,280	2011 Jul 24
A/Indiana/10/2011 (MDCK cells)*	<10	40	1,280	320	1,280	640	1,280	1,280	2011 Oct 22
A/Indiana/10/2011 (egg)*	<10	10	1,280	320	640	320	1,280	1,280	2011 Oct 22
A/Iowa/07/2011 (MDCK cells)*	<10	10	1,280	640	1,280	640	1,280	2,560	2011 Nov 14
A/Iowa/08/2011 (MDCK cells)*	<10	40	1,280	640	640	640	1,280	2,560	2011 Nov 14
A/Iowa/09/2011 (MDCK cells)*	<10	40	1,280	640	1,280	640	2560	2,560	2011 Nov 14

*Influenza A(H3N2)v, virus variant identified in humans, United States, 2011. Gray shading indicates antigenically similar viruses. †Current seasonal influenza A(H3N2) vaccine virus. ‡Reassortant virus possessing the hemagglutinin and neuraminidase genes of A/Minnesota/11/2010 and the remaining 6 genes of A/PR/8/34.

All influenza A(H3N2)v viruses tested were antigenically similar, demonstrating hemagglutination-inhibition titers with only a 2-fold difference from antiserum against other influenza A(H3N2)v viruses. These viruses were also antigenically closely related to earlier human triple-reassortant virus isolates that contained the M gene from classical SIVs (A/Wisconsin/12/2010, A/Pennsylvania/14/2010, and A/Minnesota/11/2010). All influenza A(H3N2)v viruses tested were also antigenically closely related to the proposed vaccine reassortant X-203 (*13*) between triple-reassortant A/Minnesota/11/2010 (H3N2) and A/PR/8/34 (H1N1) (Table 2).

The level of cross-protective immunity against influenza A(H3N2)v in humans previously vaccinated and/or exposed to previously circulated seasonal influenza A(H3N2) viruses is unknown. The antigenic characterization described here demonstrates that vaccination with the current trivalent influenza vaccine might not provide immune protection against influenza A(H3N2)v virus. A vaccine containing a contemporary influenza A(H3N2)v or an antigenically similar virus (such as A/Minnesota/11/2010) might be needed to elicit protective immunity.

Functional neuraminidase inhibition assays indicated that 6 influenza A(H3N2)v virus isolates were sensitive to the neuraminidase inhibitors oseltamivir and zanamivir. No genetic markers known to decrease sensitivity to neuraminidase inhibitors were found in the neuraminidase genes of all 12 influenza A(H3N2)v viruses. Similar to pandemic (H1N1) 2009 viruses, influenza A(H3N2)v viruses have genetic markers (V27A, S31N) in the M2 protein that confer resistance to the antiviral medications amantadine and rimantadine.

## Conclusions

The detection of multiple cases of human infection with influenza A(H3N2)v virus within a 5-month period in 5 US states, coupled with possible human-to-human transmission, underscores the need for continued influenza surveillance at the swine–human interface. Coordinated surveillance of human and animal influenza viruses enables rapid detection of human infections with novel influenza viruses and timely identification of new virus variants in swine. As was evident during the 2009 influenza pandemic, this information is vital for development of resources that might be needed to effectively respond to the emergence and spread of a novel influenza virus in humans.

## Supplementary Material

Technical Appendix Phylogenetic analyses of protein genes and gene sequence accession numbers for influenza virus A(H3N2)v.
