# Supplementary material for: Human Infections with Novel Reassortant Influenza A(H3N2)v Viruses, United States, 2011
Source: Emerg Infect Dis. 2012 May;18(5):834–7. doi: 10.3201/eid1805.111922 (PMC3358066; doi:10.3201/eid1805.111922)
Supplement: Technical Appendix — Phylogenetic analyses of protein genes and gene sequence accession numbers for influenza virus A(H3N2)v. [file 11-1922-Techapp_9p.pdf]

Article DOI: http://dx.doi.org/10.3201/eid1805.111922

Human Infections with Novel Reassortant Influenza A(H3N2)v Viruses, United States, 2011

Technical Appendix

Technical Appendix Figure (following pages). Phylogenetic analyses of the A) polymerase basic (PB) protein 2, B) PB1 protein, C) polymerase acidic protein, D) nucleoprotein, E) neuraminidase, and F) nonstructural protein genes of influenza A(H3N2)v virus. Sequences obtained from human isolates in the United States during 2011 are shown in red; strains from 2009 are shown in blue. Scale bars indicate number of base substitutions per site.

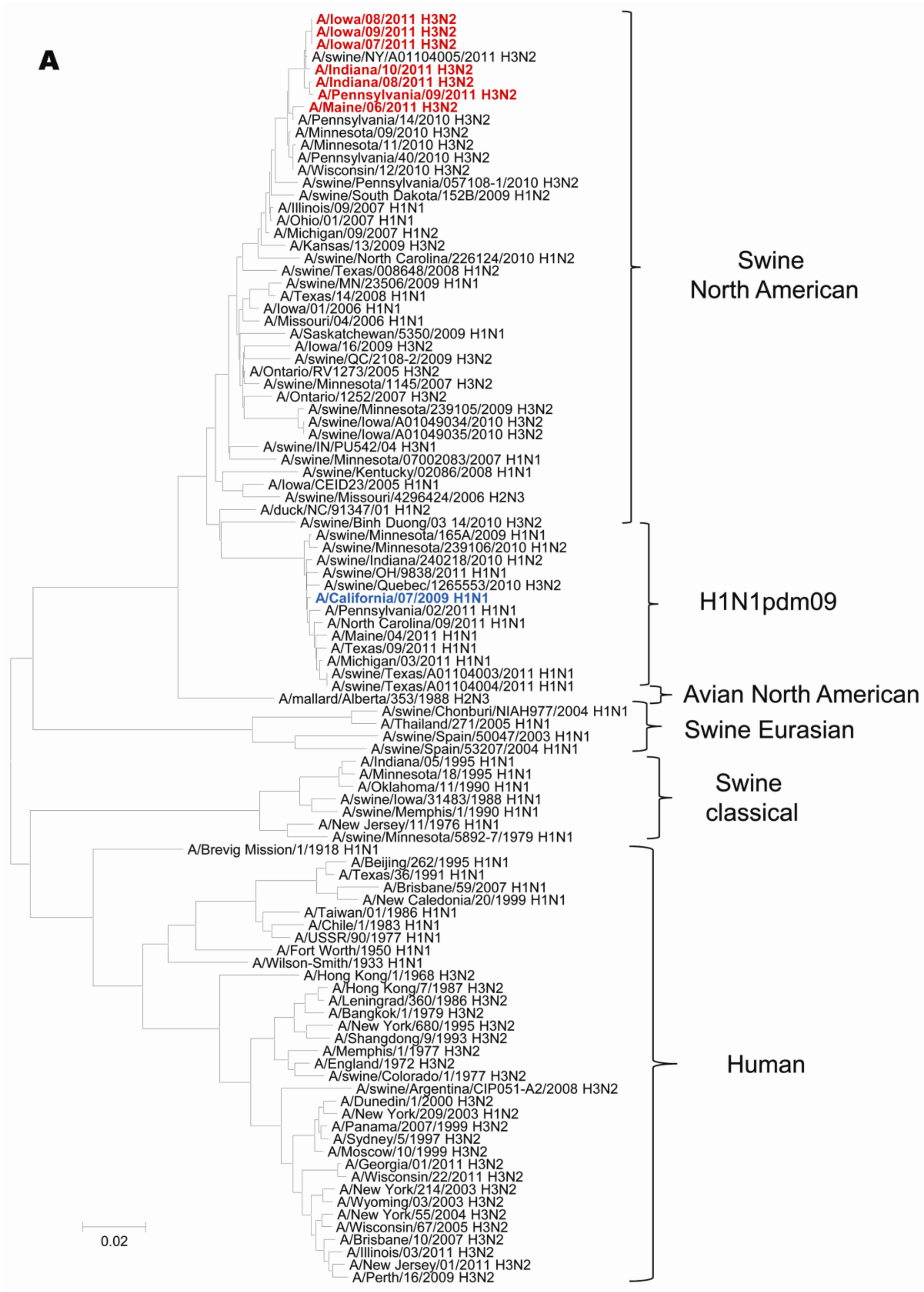
A
A/Iowa/08/2011 H3N2
A/Iowa/09/2011 H3N2
A/Iowa/07/2011 H3N2
A/swine/NY/A01104005/2011 H3N2
A/Indiana/10/2011 H3N2
A/Indiana/08/2011 H3N2
A/Pennsylvania/09/2011 H3N2
A/Maine/06/2011 H3N2
A/Pennsylvania/14/2010 H3N2
A/Minnesota/09/2010 H3N2
A/Minnesota/11/2010 H3N2
A/Pennsylvania/40/2010 H3N2
A/Wisconsin/12/2010 H3N2
A/swine/Pennsylvania/057108-1/2010 H3N2
A/swine/South Dakota/152B/2009 H1N2
A/Illinois/09/2007 H1N1
A/Ohio/01/2007 H1N1
A/Michigan/09/2007 H1N2
A/Kansas/13/2009 H3N2
A/swine/North Carolina/226124/2010 H1N2
A/swine/Texas/008648/2008 H1N2
A/swine/MN/23506/2009 H1N1
A/Texas/14/2008 H1N1
A/Iowa/01/2006 H1N1
A/Missouri/04/2006 H1N1
A/Saskatchewan/5350/2009 H1N1
A/Iowa/16/2009 H3N2
A/swine/QC/2108-2/2009 H3N2
A/Ontario/RV1273/2005 H3N2
A/swine/Minnesota/1145/2007 H3N2
A/Ontario/1252/2007 H3N2
A/swine/Minnesota/239105/2009 H3N2
A/swine/Iowa/A01049034/2010 H3N2
A/swine/Iowa/A01049035/2010 H3N2
A/swine/IN/PU542/04 H3N1
A/swine/Minnesota/07002083/2007 H1N1
A/swine/Kentucky/02086/2008 H1N1
A/Iowa/CEID23/2005 H1N1
A/swine/Missouri/4296424/2006 H2N3
A/duck/NC/91347/01 H1N2
A/swine/Binh Duong/03 14/2010 H3N2
A/swine/Minnesota/165A/2009 H1N1
A/swine/Minnesota/239106/2010 H1N2
A/swine/Indiana/240218/2010 H1N2
A/swine/OH/9838/2011 H1N1
A/swine/Quebec/1265553/2010 H3N2
A/California/07/2009 H1N1
A/Pennsylvania/02/2011 H1N1
A/North Carolina/09/2011 H1N1
A/Maine/04/2011 H1N1
A/Texas/09/2011 H1N1
A/Michigan/03/2011 H1N1
A/swine/Texas/A01104003/2011 H1N1
A/swine/Texas/A01104004/2011 H1N1
A/mallard/Alberta/353/1988 H2N3
A/swine/Chonburi/NIAH977/2004 H1N1
A/Thailand/271/2005 H1N1
A/swine/Spain/50047/2003 H1N1
A/swine/Spain/53207/2004 H1N1
A/Indiana/05/1995 H1N1
A/Minnesota/18/1995 H1N1
A/Oklahoma/11/1990 H1N1
A/swine/Iowa/31483/1988 H1N1
A/swine/Memphis/1/1990 H1N1
A/New Jersey/11/1976 H1N1
A/swine/Minnesota/5892-7/1979 H1N1
A/Brevig Mission/1/1918 H1N1
A/Beijing/262/1995 H1N1
A/Texas/36/1991 H1N1
A/Brisbane/59/2007 H1N1
A/New Caledonia/20/1999 H1N1
A/Taiwan/01/1986 H1N1
A/Chile/1/1983 H1N1
A/USSR/90/1977 H1N1
A/Fort Worth/1950 H1N1
A/Wilson-Smith/1933 H1N1
A/Hong Kong/1/1968 H3N2
A/Hong Kong/7/1987 H3N2
A/Leningrad/360/1986 H3N2
A/Bangkok/1/1979 H3N2
A/New York/680/1995 H3N2
A/Shangdong/9/1993 H3N2
A/Memphis/1/1977 H3N2
A/England/1972 H3N2
A/swine/Colorado/1/1977 H3N2
A/swine/Argentina/CIP051-A2/2008 H3N2
A/Dunedin/1/2000 H3N2
A/New York/209/2003 H1N2
A/Panama/2007/1999 H3N2
A/Sydney/5/1997 H3N2
A/Moscow/10/1999 H3N2
A/Georgia/01/2011 H3N2
A/Wisconsin/22/2011 H3N2
A/New York/214/2003 H3N2
A/Wyoming/03/2003 H3N2
A/New York/55/2004 H3N2
A/Wisconsin/67/2005 H3N2
A/Brisbane/10/2007 H3N2
A/Illinois/03/2011 H3N2
A/New Jersey/01/2011 H3N2
A/Perth/16/2009 H3N2
0.02
Swine North American
H1N1pdm09
Avian North American
Swine Eurasian
Swine classical
Human

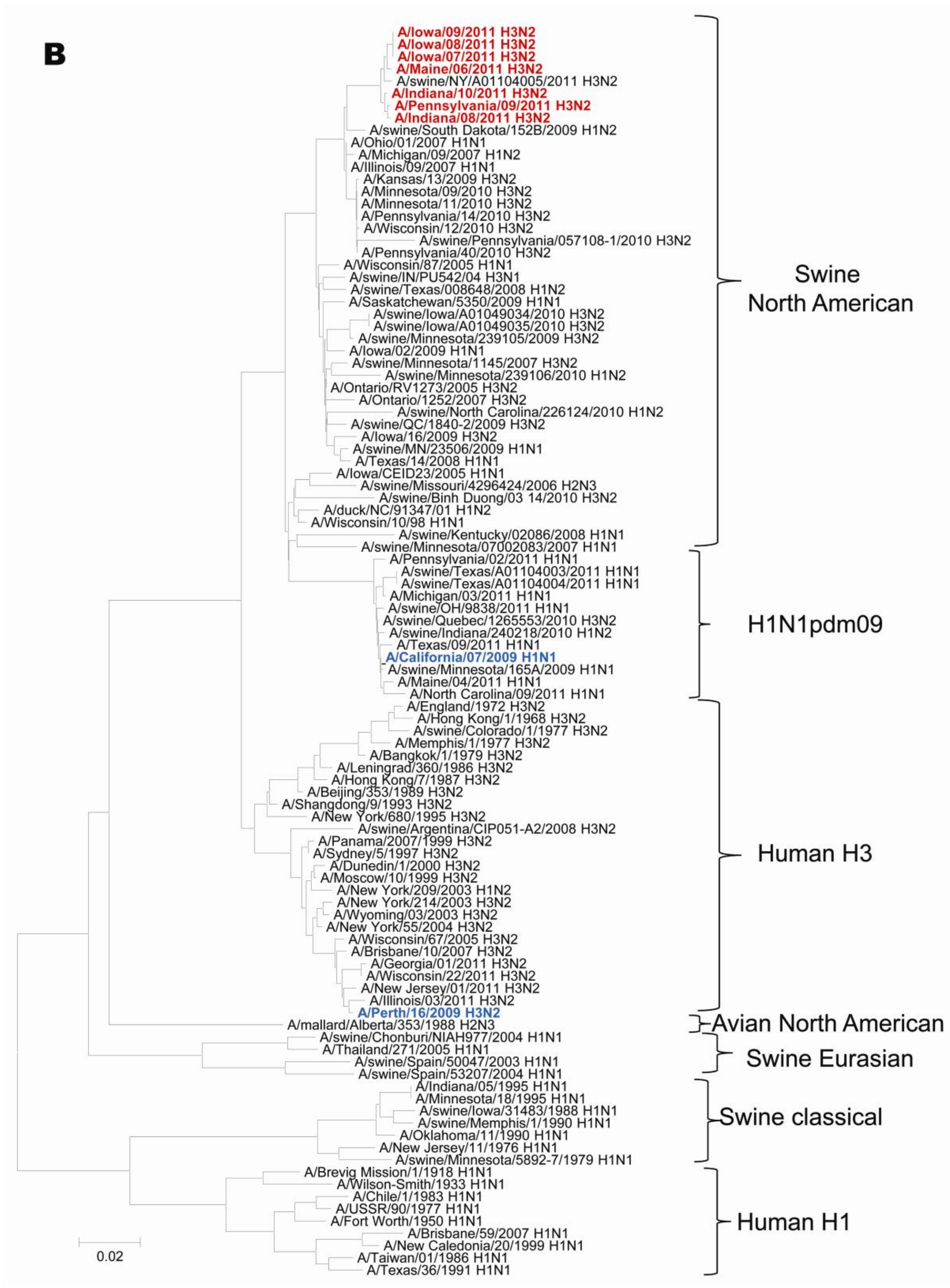
B
A/Iowa/09/2011 H3N2
A/Iowa/08/2011 H3N2
A/Iowa/07/2011 H3N2
A/Maine/06/2011 H3N2
A/swine/NY/A01104005/2011 H3N2
A/Indiana/10/2011 H3N2
A/Pennsylvania/09/2011 H3N2
A/Indiana/08/2011 H3N2
A/swine/South Dakota/152B/2009 H1N2
A/Ohio/01/2007 H1N1
A/Michigan/09/2007 H1N2
A/Illinois/09/2007 H1N1
A/Kansas/13/2009 H3N2
A/Minnesota/09/2010 H3N2
A/Minnesota/11/2010 H3N2
A/Pennsylvania/14/2010 H3N2
A/Wisconsin/12/2010 H3N2
A/swine/Pennsylvania/057108-1/2010 H3N2
A/Pennsylvania/40/2010 H3N2
A/Wisconsin/87/2005 H1N1
A/swine/IN/PU542/04 H3N1
A/swine/Texas/008648/2008 H1N2
A/Saskatchewan/5350/2009 H1N1
A/swine/Iowa/A01049034/2010 H3N2
A/swine/Iowa/A01049035/2010 H3N2
A/swine/Minnesota/239105/2009 H3N2
A/Iowa/02/2009 H1N1
A/swine/Minnesota/1145/2007 H3N2
A/swine/Minnesota/239106/2010 H1N2
A/Ontario/RV1273/2005 H3N2
A/Ontario/1252/2007 H3N2
A/swine/North Carolina/226124/2010 H1N2
A/swine/QC/1840-2/2009 H3N2
A/Iowa/16/2009 H3N2
A/swine/MN/23506/2009 H1N1
A/Texas/14/2008 H1N1
A/Iowa/CEID23/2005 H1N1
A/swine/Missouri/4296424/2006 H2N3
A/swine/Binh Duong/03 14/2010 H3N2
A/duck/NC/91347/01 H1N2
A/Wisconsin/10/98 H1N1
A/swine/Kentucky/02086/2008 H1N1
A/swine/Minnesota/07002083/2007 H1N1
Swine
North American
A/Pennsylvania/02/2011 H1N1
A/swine/Texas/A01104003/2011 H1N1
A/swine/Texas/A01104004/2011 H1N1
A/Michigan/03/2011 H1N1
A/swine/OH/9838/2011 H1N1
A/swine/Quebec/1265553/2010 H3N2
A/swine/Indiana/240218/2010 H1N2
A/Texas/09/2011 H1N1
A/California/07/2009 H1N1
A/swine/Minnesota/165A/2009 H1N1
A/Maine/04/2011 H1N1
A/North Carolina/09/2011 H1N1
H1N1pdm09
A/England/1972 H3N2
A/Hong Kong/1/1968 H3N2
A/swine/Colorado/1/1977 H3N2
A/Memphis/1/1977 H3N2
A/Bangkok/1/1979 H3N2
A/Leningrad/360/1986 H3N2
A/Hong Kong/7/1987 H3N2
A/Beijing/353/1989 H3N2
A/Shangdong/9/1993 H3N2
A/New York/680/1995 H3N2
A/swine/Argentina/CIP051-A2/2008 H3N2
A/Panama/2007/1999 H3N2
A/Sydney/5/1997 H3N2
A/Dunedin/1/2000 H3N2
A/Moscow/10/1999 H3N2
A/New York/209/2003 H1N2
A/New York/214/2003 H3N2
A/Wyoming/03/2003 H3N2
A/New York/55/2004 H3N2
A/Wisconsin/67/2005 H3N2
A/Brisbane/10/2007 H3N2
A/Georgia/01/2011 H3N2
A/Wisconsin/22/2011 H3N2
A/New Jersey/01/2011 H3N2
A/Illinois/03/2011 H3N2
A/Perth/16/2009 H3N2
Human H3
A/mallard/Alberta/353/1988 H2N3
Avian North American
A/swine/Chonburi/NIAH977/2004 H1N1
A/Thailand/271/2005 H1N1
A/swine/Spain/50047/2003 H1N1
A/swine/Spain/53207/2004 H1N1
Swine Eurasian
A/Indiana/05/1995 H1N1
A/Minnesota/18/1995 H1N1
A/swine/Iowa/31483/1988 H1N1
A/swine/Memphis/1/1990 H1N1
A/Oklahoma/11/1990 H1N1
A/New Jersey/11/1976 H1N1
A/swine/Minnesota/5892-7/1979 H1N1
Swine classical
A/Brevig Mission/1/1918 H1N1
A/Wilson-Smith/1933 H1N1
A/Chile/1/1983 H1N1
A/USSR/90/1977 H1N1
A/Fort Worth/1950 H1N1
A/Brisbane/59/2007 H1N1
A/New Caledonia/20/1999 H1N1
A/Taiwan/01/1986 H1N1
A/Texas/36/1991 H1N1
Human H1
0.02

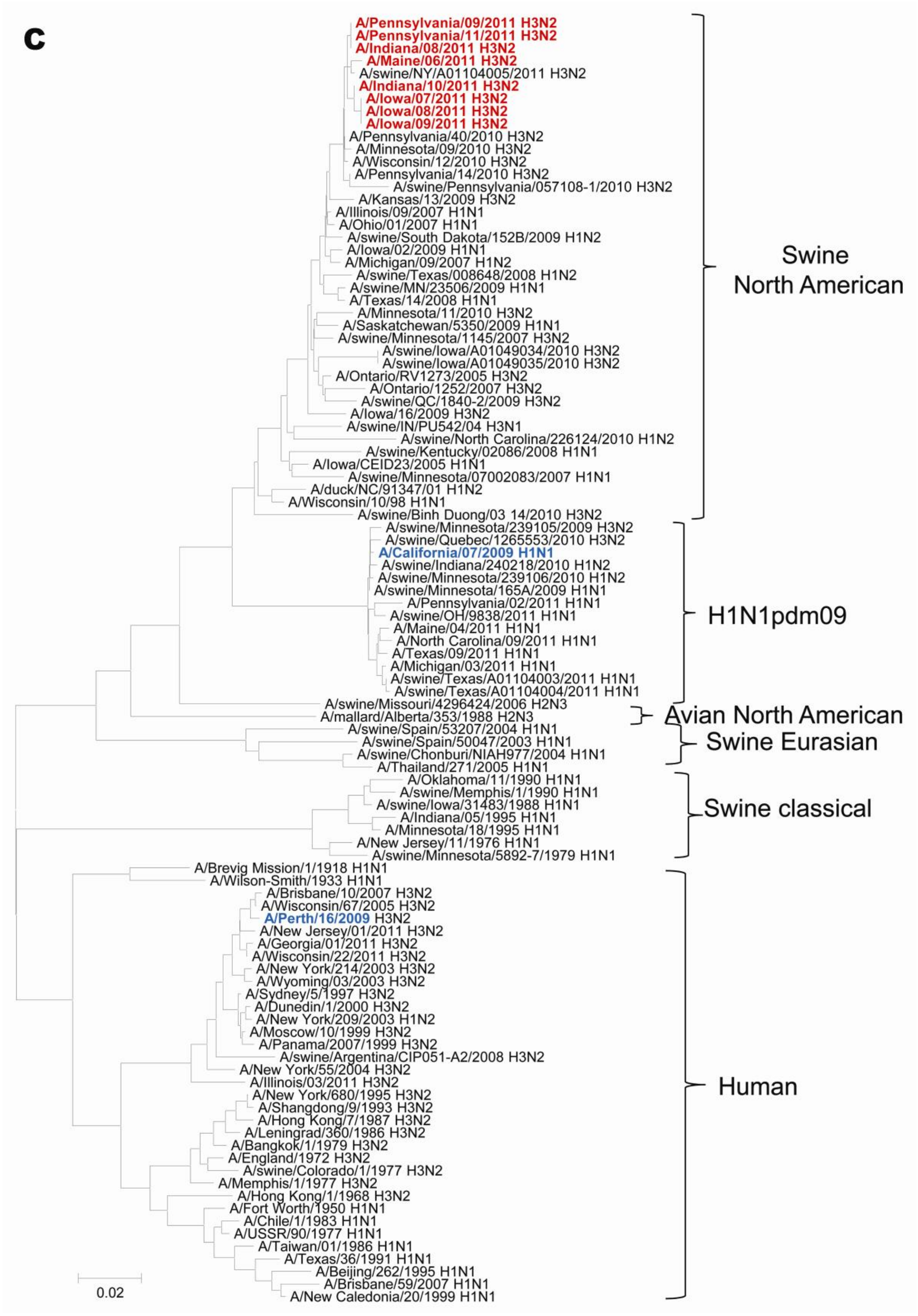
c
A/Pennsylvania/09/2011 H3N2
A/Pennsylvania/11/2011 H3N2
A/Indiana/08/2011 H3N2
A/Maine/06/2011 H3N2
A/swine/NY/A01104005/2011 H3N2
A/Indiana/10/2011 H3N2
A/Iowa/07/2011 H3N2
A/Iowa/08/2011 H3N2
A/Iowa/09/2011 H3N2
A/Pennsylvania/40/2010 H3N2
A/Minnesota/09/2010 H3N2
A/Wisconsin/12/2010 H3N2
A/Pennsylvania/14/2010 H3N2
A/swine/Pennsylvania/057108-1/2010 H3N2
A/Kansas/13/2009 H3N2
A/Illinois/09/2007 H1N1
A/Ohio/01/2007 H1N1
A/swine/South Dakota/152B/2009 H1N2
A/Iowa/02/2009 H1N1
A/Michigan/09/2007 H1N2
A/swine/Texas/008648/2008 H1N2
A/swine/MN/23506/2009 H1N1
A/Texas/14/2008 H1N1
A/Minnesota/11/2010 H3N2
A/Saskatchewan/5350/2009 H1N1
A/swine/Minnesota/1145/2007 H3N2
A/swine/Iowa/A01049034/2010 H3N2
A/swine/Iowa/A01049035/2010 H3N2
A/Ontario/RV1273/2005 H3N2
A/Ontario/1252/2007 H3N2
A/swine/QC/1840-2/2009 H3N2
A/Iowa/16/2009 H3N2
A/swine/IN/PU542/04 H3N1
A/swine/North Carolina/226124/2010 H1N2
A/swine/Kentucky/02086/2008 H1N1
A/Iowa/CEID23/2005 H1N1
A/swine/Minnesota/07002083/2007 H1N1
A/duck/NC/91347/01 H1N2
A/Wisconsin/10/98 H1N1
A/swine/Binh Duong/03 14/2010 H3N2
Swine North American
A/swine/Minnesota/239105/2009 H3N2
A/swine/Quebec/1265553/2010 H3N2
A/California/07/2009 H1N1
A/swine/Indiana/240218/2010 H1N2
A/swine/Minnesota/239106/2010 H1N2
A/swine/Minnesota/165A/2009 H1N1
A/Pennsylvania/02/2011 H1N1
A/swine/OH/9838/2011 H1N1
A/Maine/04/2011 H1N1
A/North Carolina/09/2011 H1N1
A/Texas/09/2011 H1N1
A/Michigan/03/2011 H1N1
A/swine/Texas/A01104003/2011 H1N1
A/swine/Texas/A01104004/2011 H1N1
H1N1pdm09
A/swine/Missouri/4296424/2006 H2N3
A/mallard/Alberta/353/1988 H2N3
Avian North American
A/swine/Spain/53207/2004 H1N1
A/swine/Spain/50047/2003 H1N1
A/swine/Chonburi/NIAH977/2004 H1N1
A/Thailand/271/2005 H1N1
Swine Eurasian
A/Oklahoma/11/1990 H1N1
A/swine/Memphis/1/1990 H1N1
A/swine/Iowa/31483/1988 H1N1
A/Indiana/05/1995 H1N1
A/Minnesota/18/1995 H1N1
A/New Jersey/11/1976 H1N1
A/swine/Minnesota/5892-7/1979 H1N1
Swine classical
A/Brevig Mission/1/1918 H1N1
A/Wilson-Smith/1933 H1N1
A/Brisbane/10/2007 H3N2
A/Wisconsin/67/2005 H3N2
A/Perth/16/2009 H3N2
A/New Jersey/01/2011 H3N2
A/Georgia/01/2011 H3N2
A/Wisconsin/22/2011 H3N2
A/New York/214/2003 H3N2
A/Wyoming/03/2003 H3N2
A/Sydney/5/1997 H3N2
A/Dunedin/1/2000 H3N2
A/New York/209/2003 H1N2
A/Moscow/10/1999 H3N2
A/Panama/2007/1999 H3N2
A/swine/Argentina/CIP051-A2/2008 H3N2
A/New York/55/2004 H3N2
A/Illinois/03/2011 H3N2
A/New York/680/1995 H3N2
A/Shangdong/9/1993 H3N2
A/Hong Kong/7/1987 H3N2
A/Leningrad/360/1986 H3N2
A/Bangkok/1/1979 H3N2
A/England/1972 H3N2
A/swine/Colorado/1/1977 H3N2
A/Memphis/1/1977 H3N2
A/Hong Kong/1/1968 H3N2
A/Fort Worth/1950 H1N1
A/Chile/1/1983 H1N1
A/USSR/90/1977 H1N1
A/Taiwan/01/1986 H1N1
A/Texas/36/1991 H1N1
A/Beijing/262/1995 H1N1
A/Brisbane/59/2007 H1N1
A/New Caledonia/20/1999 H1N1
Human
0.02

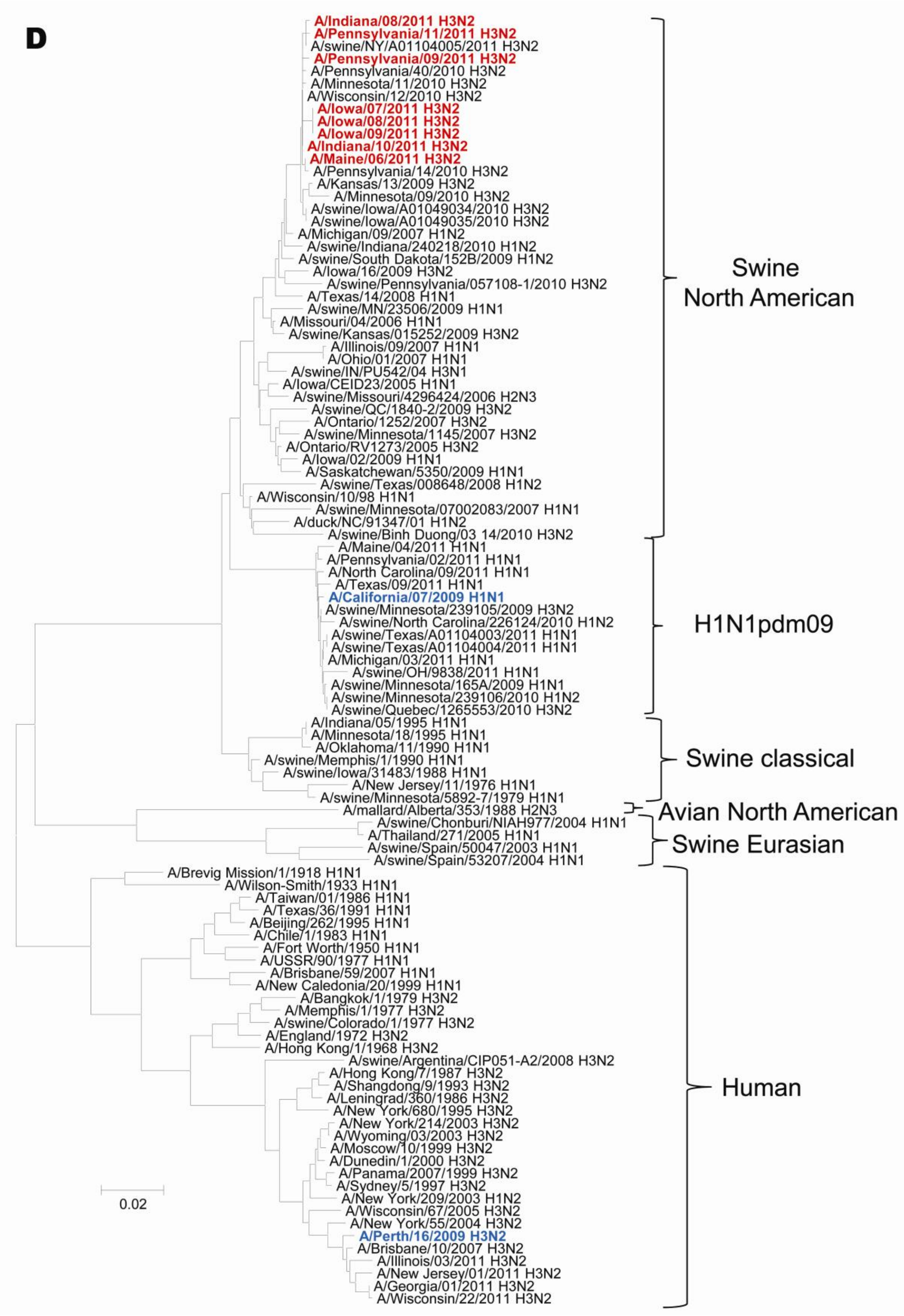
D
A/Indiana/08/2011 H3N2
A/Pennsylvania/11/2011 H3N2
A/swine/NY/A01104005/2011 H3N2
A/Pennsylvania/09/2011 H3N2
A/Pennsylvania/40/2010 H3N2
A/Minnesota/11/2010 H3N2
A/Wisconsin/12/2010 H3N2
A/Iowa/07/2011 H3N2
A/Iowa/08/2011 H3N2
A/Iowa/09/2011 H3N2
A/Indiana/10/2011 H3N2
A/Maine/06/2011 H3N2
A/Pennsylvania/14/2010 H3N2
A/Kansas/13/2009 H3N2
A/Minnesota/09/2010 H3N2
A/swine/Iowa/A01049034/2010 H3N2
A/swine/Iowa/A01049035/2010 H3N2
A/Michigan/09/2007 H1N2
A/swine/Indiana/240218/2010 H1N2
A/swine/South Dakota/152B/2009 H1N2
A/Iowa/16/2009 H3N2
A/swine/Pennsylvania/057108-1/2010 H3N2
A/Texas/14/2008 H1N1
A/swine/MN/23506/2009 H1N1
A/Missouri/04/2006 H1N1
A/swine/Kansas/015252/2009 H3N2
A/Illinois/09/2007 H1N1
A/Ohio/01/2007 H1N1
A/swine/IN/PU542/04 H3N1
A/Iowa/CEID23/2005 H1N1
A/swine/Missouri/4296424/2006 H2N3
A/swine/QC/1840-2/2009 H3N2
A/Ontario/1252/2007 H3N2
A/swine/Minnesota/1145/2007 H3N2
A/Ontario/RV1273/2005 H3N2
A/Iowa/02/2009 H1N1
A/Saskatchewan/5350/2009 H1N1
A/swine/Texas/008648/2008 H1N2
A/Wisconsin/10/98 H1N1
A/swine/Minnesota/07002083/2007 H1N1
A/duck/NC/91347/01 H1N2
A/swine/Binh Duong/03 14/2010 H3N2
Swine
North American
A/Maine/04/2011 H1N1
A/Pennsylvania/02/2011 H1N1
A/North Carolina/09/2011 H1N1
A/Texas/09/2011 H1N1
A/California/07/2009 H1N1
A/swine/Minnesota/239105/2009 H3N2
A/swine/North Carolina/226124/2010 H1N2
A/swine/Texas/A01104003/2011 H1N1
A/swine/Texas/A01104004/2011 H1N1
A/Michigan/03/2011 H1N1
A/swine/OH/9838/2011 H1N1
A/swine/Minnesota/165A/2009 H1N1
A/swine/Minnesota/239106/2010 H1N2
A/swine/Quebec/1265553/2010 H3N2
H1N1pdm09
A/Indiana/05/1995 H1N1
A/Minnesota/18/1995 H1N1
A/Oklahoma/11/1990 H1N1
A/swine/Memphis/1/1990 H1N1
A/swine/Iowa/31483/1988 H1N1
A/New Jersey/11/1976 H1N1
A/swine/Minnesota/5892-7/1979 H1N1
Swine classical
A/mallard/Alberta/353/1988 H2N3
Avian North American
A/swine/Chonburi/NIAH977/2004 H1N1
A/Thailand/271/2005 H1N1
A/swine/Spain/50047/2003 H1N1
A/swine/Spain/53207/2004 H1N1
Swine Eurasian
A/Brevig Mission/1/1918 H1N1
A/Wilson-Smith/1933 H1N1
A/Taiwan/01/1986 H1N1
A/Texas/36/1991 H1N1
A/Beijing/262/1995 H1N1
A/Chile/1/1983 H1N1
A/Fort Worth/1950 H1N1
A/USSR/90/1977 H1N1
A/Brisbane/59/2007 H1N1
A/New Caledonia/20/1999 H1N1
A/Bangkok/1/1979 H3N2
A/Memphis/1/1977 H3N2
A/swine/Colorado/1/1977 H3N2
A/England/1972 H3N2
A/Hong Kong/1/1968 H3N2
A/swine/Argentina/CIP051-A2/2008 H3N2
A/Hong Kong/7/1987 H3N2
A/Shangdong/9/1993 H3N2
A/Leningrad/360/1986 H3N2
A/New York/680/1995 H3N2
A/New York/214/2003 H3N2
A/Wyoming/03/2003 H3N2
A/Moscow/10/1999 H3N2
A/Dunedin/1/2000 H3N2
A/Panama/2007/1999 H3N2
A/Sydney/5/1997 H3N2
A/New York/209/2003 H1N2
A/Wisconsin/67/2005 H3N2
A/New York/55/2004 H3N2
A/Perth/16/2009 H3N2
A/Brisbane/10/2007 H3N2
A/Illinois/03/2011 H3N2
A/New Jersey/01/2011 H3N2
A/Georgia/01/2011 H3N2
A/Wisconsin/22/2011 H3N2
Human
0.02

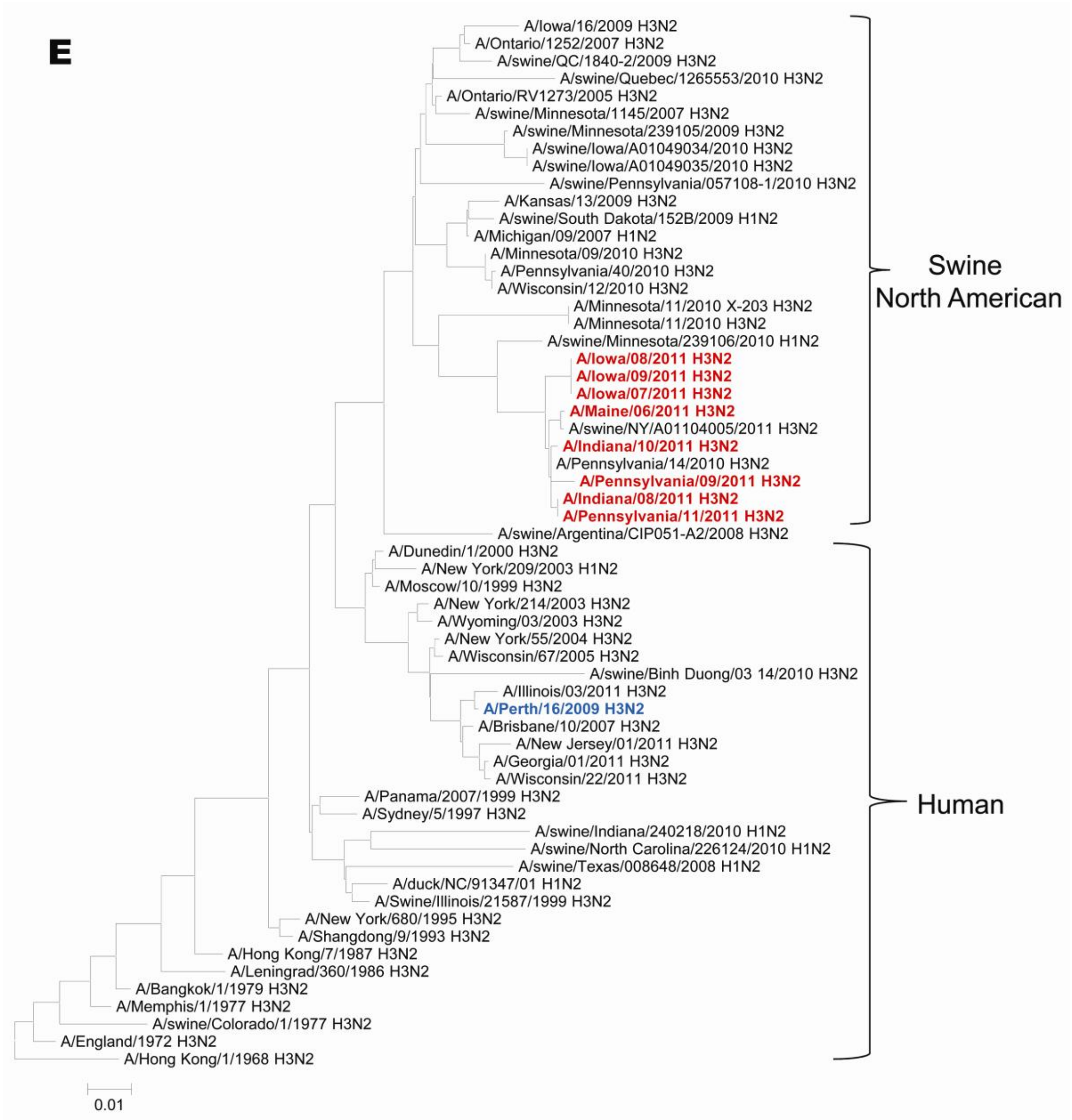
E
A/Iowa/16/2009 H3N2
A/Ontario/1252/2007 H3N2
A/swine/QC/1840-2/2009 H3N2
A/swine/Quebec/1265553/2010 H3N2
A/Ontario/RV1273/2005 H3N2
A/swine/Minnesota/1145/2007 H3N2
A/swine/Minnesota/239105/2009 H3N2
A/swine/Iowa/A01049034/2010 H3N2
A/swine/Iowa/A01049035/2010 H3N2
A/swine/Pennsylvania/057108-1/2010 H3N2
A/Kansas/13/2009 H3N2
A/swine/South Dakota/152B/2009 H1N2
A/Michigan/09/2007 H1N2
A/Minnesota/09/2010 H3N2
A/Pennsylvania/40/2010 H3N2
A/Wisconsin/12/2010 H3N2
A/Minnesota/11/2010 X-203 H3N2
A/Minnesota/11/2010 H3N2
A/swine/Minnesota/239106/2010 H1N2
A/Iowa/08/2011 H3N2
A/Iowa/09/2011 H3N2
A/Iowa/07/2011 H3N2
A/Maine/06/2011 H3N2
A/swine/NY/A01104005/2011 H3N2
A/Indiana/10/2011 H3N2
A/Pennsylvania/14/2010 H3N2
A/Pennsylvania/09/2011 H3N2
A/Indiana/08/2011 H3N2
A/Pennsylvania/11/2011 H3N2
A/swine/Argentina/CIP051-A2/2008 H3N2
A/Dunedin/1/2000 H3N2
A/New York/209/2003 H1N2
A/Moscow/10/1999 H3N2
A/New York/214/2003 H3N2
A/Wyoming/03/2003 H3N2
A/New York/55/2004 H3N2
A/Wisconsin/67/2005 H3N2
A/swine/Binh Duong/03 14/2010 H3N2
A/Illinois/03/2011 H3N2
A/Perth/16/2009 H3N2
A/Brisbane/10/2007 H3N2
A/New Jersey/01/2011 H3N2
A/Georgia/01/2011 H3N2
A/Wisconsin/22/2011 H3N2
A/Panama/2007/1999 H3N2
A/Sydney/5/1997 H3N2
A/swine/Indiana/240218/2010 H1N2
A/swine/North Carolina/226124/2010 H1N2
A/swine/Texas/008648/2008 H1N2
A/duck/NC/91347/01 H1N2
A/Swine/Illinois/21587/1999 H3N2
A/New York/680/1995 H3N2
A/Shangdong/9/1993 H3N2
A/Hong Kong/7/1987 H3N2
A/Leningrad/360/1986 H3N2
A/Bangkok/1/1979 H3N2
A/Memphis/1/1977 H3N2
A/swine/Colorado/1/1977 H3N2
A/England/1972 H3N2
A/Hong Kong/1/1968 H3N2
Swine North American
Human
0.01

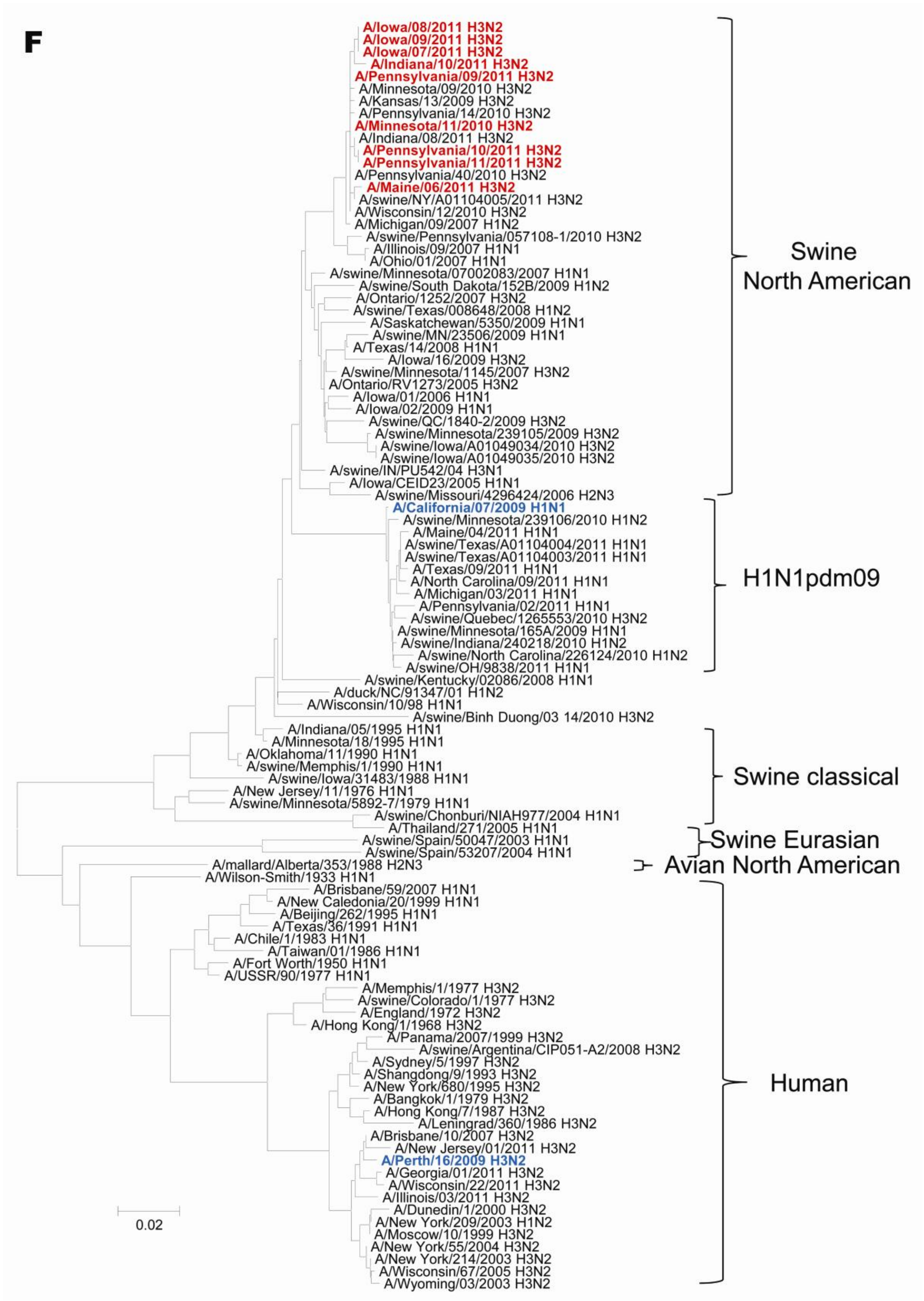
F
A/Iowa/08/2011 H3N2
A/Iowa/09/2011 H3N2
A/Iowa/07/2011 H3N2
A/Indiana/10/2011 H3N2
A/Pennsylvania/09/2011 H3N2
A/Minnesota/09/2010 H3N2
A/Kansas/13/2009 H3N2
A/Pennsylvania/14/2010 H3N2
A/Minnesota/11/2010 H3N2
A/Indiana/08/2011 H3N2
A/Pennsylvania/10/2011 H3N2
A/Pennsylvania/11/2011 H3N2
A/Pennsylvania/40/2010 H3N2
A/Maine/06/2011 H3N2
A/swine/NY/A01104005/2011 H3N2
A/Wisconsin/12/2010 H3N2
A/Michigan/09/2007 H1N2
A/swine/Pennsylvania/057108-1/2010 H3N2
A/Illinois/09/2007 H1N1
A/Ohio/01/2007 H1N1
A/swine/Minnesota/07002083/2007 H1N2
A/swine/South Dakota/152B/2009 H1N2
A/Ontario/1252/2007 H3N2
A/swine/Texas/008648/2008 H1N2
A/Saskatchewan/5350/2009 H1N1
A/swine/MN/23506/2009 H1N1
A/Texas/14/2008 H1N1
A/Iowa/16/2009 H3N2
A/swine/Minnesota/1145/2007 H3N2
A/Ontario/RV1273/2005 H3N2
A/Iowa/01/2006 H1N1
A/Iowa/02/2009 H1N1
A/swine/QC/1840-2/2009 H3N2
A/swine/Minnesota/239105/2009 H3N2
A/swine/Iowa/A01049034/2010 H3N2
A/swine/Iowa/A01049035/2010 H3N2
A/swine/IN/PU542/04 H3N1
A/Iowa/CEID23/2005 H1N1
A/swine/Missouri/4296424/2006 H2N3
Swine
North American
A/California/07/2009 H1N1
A/swine/Minnesota/239106/2010 H1N2
A/Maine/04/2011 H1N1
A/swine/Texas/A01104004/2011 H1N1
A/swine/Texas/A01104003/2011 H1N1
A/Texas/09/2011 H1N1
A/North Carolina/09/2011 H1N1
A/Michigan/03/2011 H1N1
A/Pennsylvania/02/2011 H1N1
A/swine/Quebec/1265553/2010 H3N2
A/swine/Minnesota/165A/2009 H1N1
A/swine/Indiana/240218/2010 H1N2
A/swine/North Carolina/226124/2010 H1N2
A/swine/OH/9838/2011 H1N1
H1N1pdm09
A/swine/Kentucky/02086/2008 H1N1
A/duck/NC/91347/01 H1N2
A/Wisconsin/10/98 H1N1
A/swine/Binh Duong/03 14/2010 H3N2
A/Indiana/05/1995 H1N1
A/Minnesota/18/1995 H1N1
A/Oklahoma/11/1990 H1N1
A/swine/Memphis/1/1990 H1N1
A/swine/Iowa/31483/1988 H1N1
A/New Jersey/11/1976 H1N1
A/swine/Minnesota/5892-7/1979 H1N1
A/swine/Chonburi/NIAH977/2004 H1N1
A/Thailand/271/2005 H1N1
Swine classical
A/swine/Spain/50047/2003 H1N1
A/swine/Spain/53207/2004 H1N1
Swine Eurasian
A/mallard/Alberta/353/1988 H2N3
Avian North American
A/Wilson-Smith/1933 H1N1
A/Brisbane/59/2007 H1N1
A/New Caledonia/20/1999 H1N1
A/Beijing/262/1995 H1N1
A/Texas/36/1991 H1N1
A/Chile/1/1983 H1N1
A/Taiwan/01/1986 H1N1
A/Fort Worth/1950 H1N1
A/USSR/90/1977 H1N1
A/Memphis/1/1977 H3N2
A/swine/Colorado/1/1977 H3N2
A/England/1972 H3N2
A/Hong Kong/1/1968 H3N2
A/Panama/2007/1999 H3N2
A/swine/Argentina/CIP051-A2/2008 H3N2
A/Sydney/5/1997 H3N2
A/Shangdong/9/1993 H3N2
A/New York/680/1995 H3N2
A/Bangkok/1/1979 H3N2
A/Hong Kong/7/1987 H3N2
A/Leningrad/360/1986 H3N2
A/Brisbane/10/2007 H3N2
A/New Jersey/01/2011 H3N2
A/Perth/16/2009 H3N2
A/Georgia/01/2011 H3N2
A/Wisconsin/22/2011 H3N2
A/Illinois/03/2011 H3N2
A/Dunedin/1/2000 H3N2
A/New York/209/2003 H1N2
A/Moscow/10/1999 H3N2
A/New York/55/2004 H3N2
A/New York/214/2003 H3N2
A/Wisconsin/67/2005 H3N2
A/Wyoming/03/2003 H3N2
Human
0.02

Technical Appendix Table. Gene sequence accession numbers for influenza A (H3N2)v, from The Global Initiative on Sharing All Influenza Data

Segment ID	Segment	Collection date	Isolate name	Originating laboratory†
EPI344405	HA	2011 Jul 27	A/Indiana/08/2011	Indiana State Department of Health Laboratories
EPI335629	HA	2011 Jul 27	A/Indiana/08/2011	Indiana State Department of Health Laboratories
EPI333152	HA	2011 Jul 27	A/Indiana/08/2011	Indiana State Department of Health Laboratories
EPI344400	M	2011 Jul 27	A/Indiana/08/2011	Indiana State Department of Health Laboratories
EPI335632	M	2011 Jul 27	A/Indiana/08/2011	Indiana State Department of Health Laboratories
EPI333148	M	2011 Jul 27	A/Indiana/08/2011	Indiana State Department of Health Laboratories
EPI344404	NA	2011 Jul 27	A/Indiana/08/2011	Indiana State Department of Health Laboratories
EPI335631	NA	2011 Jul 27	A/Indiana/08/2011	Indiana State Department of Health Laboratories
EPI333151	NA	2011 Jul 27	A/Indiana/08/2011	Indiana State Department of Health Laboratories
EPI344398	NP	2011 Jul 27	A/Indiana/08/2011	Indiana State Department of Health Laboratories
EPI335630	NP	2011 Jul 27	A/Indiana/08/2011	Indiana State Department of Health Laboratories
EPI333146	NP	2011 Jul 27	A/Indiana/08/2011	Indiana State Department of Health Laboratories
EPI344399	NS	2011 Jul 27	A/Indiana/08/2011	Indiana State Department of Health Laboratories
EPI335633	NS	2011 Jul 27	A/Indiana/08/2011	Indiana State Department of Health Laboratories
EPI333147	NS	2011 Jul 27	A/Indiana/08/2011	Indiana State Department of Health Laboratories
EPI344401	PA	2011 Jul 27	A/Indiana/08/2011	Indiana State Department of Health Laboratories
EPI335628	PA	2011 Jul 27	A/Indiana/08/2011	Indiana State Department of Health Laboratories
EPI333149	PA	2011 Jul 27	A/Indiana/08/2011	Indiana State Department of Health Laboratories
EPI344403	PB1	2011 Jul 27	A/Indiana/08/2011	Indiana State Department of Health Laboratories
EPI335627	PB1	2011 Jul 27	A/Indiana/08/2011	Indiana State Department of Health Laboratories
EPI333150	PB1	2011 Jul 27	A/Indiana/08/2011	Indiana State Department of Health Laboratories
EPI344402	PB2	2011 Jul 27	A/Indiana/08/2011	Indiana State Department of Health Laboratories
EPI335626	PB2	2011 Jul 27	A/Indiana/08/2011	Indiana State Department of Health Laboratories
EPI333194	PB2	2011 Jul 27	A/Indiana/08/2011	Indiana State Department of Health Laboratories
EPI344397	HA	2011 Oct 22	A/Indiana/10/2011	Indiana State Department of Health Laboratories
EPI344389	HA	2011 Oct 22	A/Indiana/10/2011	Indiana State Department of Health Laboratories
EPI340984	HA	2011 Oct 22	A/Indiana/10/2011	Indiana State Department of Health Laboratories
EPI344392	M	2011 Oct 22	A/Indiana/10/2011	Indiana State Department of Health Laboratories
EPI344385	M	2011 Oct 22	A/Indiana/10/2011	Indiana State Department of Health Laboratories
EPI340979	M	2011 Oct 22	A/Indiana/10/2011	Indiana State Department of Health Laboratories
EPI344396	NA	2011 Oct 22	A/Indiana/10/2011	Indiana State Department of Health Laboratories
EPI344388	NA	2011 Oct 22	A/Indiana/10/2011	Indiana State Department of Health Laboratories
EPI340983	NA	2011 Oct 22	A/Indiana/10/2011	Indiana State Department of Health Laboratories
EPI344390	NP	2011 Oct 22	A/Indiana/10/2011	Indiana State Department of Health Laboratories
EPI344383	NP	2011 Oct 22	A/Indiana/10/2011	Indiana State Department of Health Laboratories
EPI340977	NP	2011 Oct 22	A/Indiana/10/2011	Indiana State Department of Health Laboratories
EPI344391	NS	2011 Oct 22	A/Indiana/10/2011	Indiana State Department of Health Laboratories
EPI344384	NS	2011 Oct 22	A/Indiana/10/2011	Indiana State Department of Health Laboratories
EPI340978	NS	2011 Oct 22	A/Indiana/10/2011	Indiana State Department of Health Laboratories
EPI344393	PA	2011 Oct 22	A/Indiana/10/2011	Indiana State Department of Health Laboratories
EPI344386	PA	2011 Oct 22	A/Indiana/10/2011	Indiana State Department of Health Laboratories
EPI340980	PA	2011 Oct 22	A/Indiana/10/2011	Indiana State Department of Health Laboratories
EPI344395	PB1	2011 Oct 22	A/Indiana/10/2011	Indiana State Department of Health Laboratories
EPI344387	PB1	2011 Oct 22	A/Indiana/10/2011	Indiana State Department of Health Laboratories
EPI340982	PB1	2011 Oct 22	A/Indiana/10/2011	Indiana State Department of Health Laboratories
EPI344394	PB2	2011 Oct 22	A/Indiana/10/2011	Indiana State Department of Health Laboratories
EPI345358	PB2	2011 Oct 22	A/Indiana/10/2011	Indiana State Department of Health Laboratories
EPI340981	PB2	2011 Oct 22	A/Indiana/10/2011	Indiana State Department of Health Laboratories
EPI342688	HA	2011 Nov 14	A/Iowa/07/2011	Iowa State Hygienic Laboratory
EPI342683	M	2011 Nov 14	A/Iowa/07/2011	Iowa State Hygienic Laboratory
EPI342687	NA	2011 Nov 14	A/Iowa/07/2011	Iowa State Hygienic Laboratory
EPI342681	NP	2011 Nov 14	A/Iowa/07/2011	Iowa State Hygienic Laboratory
EPI342682	NS	2011 Nov 14	A/Iowa/07/2011	Iowa State Hygienic Laboratory
EPI342684	PA	2011 Nov 14	A/Iowa/07/2011	Iowa State Hygienic Laboratory
EPI342686	PB1	2011 Nov 14	A/Iowa/07/2011	Iowa State Hygienic Laboratory
EPI342685	PB2	2011 Nov 14	A/Iowa/07/2011	Iowa State Hygienic Laboratory
EPI342696	HA	2011 Nov 14	A/Iowa/08/2011	Iowa State Hygienic Laboratory
EPI342691	M	2011 Nov 14	A/Iowa/08/2011	Iowa State Hygienic Laboratory
EPI342695	NA	2011 Nov 14	A/Iowa/08/2011	Iowa State Hygienic Laboratory
EPI342689	NP	2011 Nov 14	A/Iowa/08/2011	Iowa State Hygienic Laboratory
EPI342690	NS	2011 Nov 14	A/Iowa/08/2011	Iowa State Hygienic Laboratory
EPI342692	PA	2011 Nov 14	A/Iowa/08/2011	Iowa State Hygienic Laboratory
EPI342694	PB1	2011 Nov 14	A/Iowa/08/2011	Iowa State Hygienic Laboratory

Segment ID	Segment	Collection date	Isolate name	Originating laboratory†
EPI342693	PB2	2011 Nov 14	A/Iowa/08/2011	Iowa State Hygienic Laboratory
EPI342704	HA	2011 Nov 14	A/Iowa/09/2011	Iowa State Hygienic Laboratory
EPI342699	M	2011 Nov 14	A/Iowa/09/2011	Iowa State Hygienic Laboratory
EPI342703	NA	2011 Nov 14	A/Iowa/09/2011	Iowa State Hygienic Laboratory
EPI342697	NP	2011 Nov 14	A/Iowa/09/2011	Iowa State Hygienic Laboratory
EPI342698	NS	2011 Nov 14	A/Iowa/09/2011	Iowa State Hygienic Laboratory
EPI342700	PA	2011 Nov 14	A/Iowa/09/2011	Iowa State Hygienic Laboratory
EPI342702	PB1	2011 Nov 14	A/Iowa/09/2011	Iowa State Hygienic Laboratory
EPI342701	PB2	2011 Nov 14	A/Iowa/09/2011	Iowa State Hygienic Laboratory
EPI339171	HA	2011 Oct 10	A/Maine/06/2011	Maine Health and Environmental Testing Laboratory
EPI339166	M	2011 Oct 10	A/Maine/06/2011	Maine Health and Environmental Testing Laboratory
EPI339170	NA	2011 Oct 10	A/Maine/06/2011	Maine Health and Environmental Testing Laboratory
EPI339164	NP	2011 Oct 10	A/Maine/06/2011	Maine Health and Environmental Testing Laboratory
EPI339165	NS	2011 Oct 10	A/Maine/06/2011	Maine Health and Environmental Testing Laboratory
EPI339167	PA	2011 Oct 10	A/Maine/06/2011	Maine Health and Environmental Testing Laboratory
EPI339169	PB1	2011 Oct 10	A/Maine/06/2011	Maine Health and Environmental Testing Laboratory
EPI339168	PB2	2011 Oct 10	A/Maine/06/2011	Maine Health and Environmental Testing Laboratory
EPI340976	HA	2011 Oct 24	A/Maine/07/2011	Maine Health and Environmental Testing Laboratory
EPI340975	M	2011 Oct 24	A/Maine/07/2011	Maine Health and Environmental Testing Laboratory
EPI340974	NP	2011 Oct 24	A/Maine/07/2011	Maine Health and Environmental Testing Laboratory
EPI341331	NS	2011 Oct 24	A/Maine/07/2011	Maine Health and Environmental Testing Laboratory
EPI335610	HA	2011 Aug 20	A/Pennsylvania/09/2011	Pennsylvania Department of Health
EPI335613	M	2011 Aug 20	A/Pennsylvania/09/2011	Pennsylvania Department of Health
EPI335612	NA	2011 Aug 20	A/Pennsylvania/09/2011	Pennsylvania Department of Health
EPI335611	NP	2011 Aug 20	A/Pennsylvania/09/2011	Pennsylvania Department of Health
EPI335614	NS	2011 Aug 20	A/Pennsylvania/09/2011	Pennsylvania Department of Health
EPI335609	PA	2011 Aug 20	A/Pennsylvania/09/2011	Pennsylvania Department of Health
EPI335608	PB1	2011 Aug 20	A/Pennsylvania/09/2011	Pennsylvania Department of Health
EPI335607	PB2	2011 Aug 20	A/Pennsylvania/09/2011	Pennsylvania Department of Health
EPI335623	HA	2011 Aug 26	A/Pennsylvania/10/2011	Pennsylvania Department of Health
EPI335624	M	2011 Aug 26	A/Pennsylvania/10/2011	Pennsylvania Department of Health
EPI335696	NA	2011 Aug 26	A/Pennsylvania/10/2011	Pennsylvania Department of Health
EPI335625	NS	2011 Aug 26	A/Pennsylvania/10/2011	Pennsylvania Department of Health
EPI335618	HA	2011 Aug 25	A/Pennsylvania/11/2011	University of Pittsburgh Medical Center Microbiology Lab
EPI335621	M	2011 Aug 25	A/Pennsylvania/11/2011	University of Pittsburgh Medical Center Microbiology Lab
EPI335620	NA	2011 Aug 25	A/Pennsylvania/11/2011	University of Pittsburgh Medical Center Microbiology Lab
EPI335619	NP	2011 Aug 25	A/Pennsylvania/11/2011	University of Pittsburgh Medical Center Microbiology Lab
EPI335622	NS	2011 Aug 25	A/Pennsylvania/11/2011	University of Pittsburgh Medical Center Microbiology Lab
EPI335617	PA	2011 Aug 25	A/Pennsylvania/11/2011	University of Pittsburgh Medical Center Microbiology Lab
EPI335616	PB1	2011 Aug 25	A/Pennsylvania/11/2011	University of Pittsburgh Medical Center Microbiology Lab
EPI335615	PB2	2011 Aug 25	A/Pennsylvania/11/2011	University of Pittsburgh Medical Center Microbiology Lab
EPI346486	NP	2011 Nov 21	A/West Virginia/06/2011	West Virginia Office of Laboratory Services
EPI346487	NS	2011 Nov 21	A/West Virginia/06/2011	West Virginia Office of Laboratory Services
EPI346488	M	2011 Nov 21	A/West Virginia/06/2011	West Virginia Office of Laboratory Services
EPI346489	PA	2011 Nov 21	A/West Virginia/06/2011	West Virginia Office of Laboratory Services
EPI346490	PB2	2011 Nov 21	A/West Virginia/06/2011	West Virginia Office of Laboratory Services
EPI346491	PB1	2011 Nov 21	A/West Virginia/06/2011	West Virginia Office of Laboratory Services
EPI346492	NA	2011 Nov 21	A/West Virginia/06/2011	West Virginia Office of Laboratory Services
EPI346493	HA	2011 Nov 21	A/West Virginia/06/2011	West Virginia Office of Laboratory Services
EPI346921	HA	2011 Dec 07	A/West Virginia/07/2011	West Virginia Office of Laboratory Services
EPI346922	NA	2011 Dec 07	A/West Virginia/07/2011	West Virginia Office of Laboratory Services
EPI346924	M	2011 Dec 07	A/West Virginia/07/2011	West Virginia Office of Laboratory Services

*HA, hemagglutinin; M, matrix protein; NA, neuraminidase; NP, nucleocapsid protein; NS, nonstructural protein; PA, polymerase acidic protein; PB, polymerase basic protein.

†Submitting laboratory for all samples was the Centers for Disease Control and Prevention.
